# Bovine Meat and Milk Factor (BMMF) Protein Is Expressed in Macrophages Spread Widely over the Mucosa of Colorectal Cancer Patients

**DOI:** 10.3390/cells14060455

**Published:** 2025-03-19

**Authors:** Sumen Siqin, Ekaterina Nikitina, Mohammad Rahbari, Claudia Ernst, Damir Krunic, Emrullah Birgin, Claudia Tessmer, Ilse Hofmann, Nuh Rahbari, Timo Bund

**Affiliations:** 1 Division of Episomal-Persistent DNA in Cancer- and Chronic Diseases, German Cancer Research Center (DKFZ), 69120 Heidelberg, Germany; 2 Division of Chronic Inflammation and Cancer, German Cancer Research Center (DKFZ), 69120 Heidelberg, Germany; 3 Department of Surgery, University Hospital Mannheim, Medical Faculty Mannheim, University of Heidelberg, 68167 Mannheim, Germany; 4 Faculty of Medicine, Institute for Interdisciplinary Research on Cancer Metabolism and Chronic Inflammation, M3-Research Center for Malignome, Metabolome and Microbiome, University of Tuebingen, 72076 Tuebingen, Germany; 5 Light Microscopy Facility, German Cancer Research Center (DKFZ), 69120 Heidelberg, Germany; 6 Clinic for General and Visceral Surgery, University Hospital Ulm, 89081 Ulm, Germany; 7 Core Facility Antibodies, German Cancer Research Center (DKFZ), 69120 Heidelberg, Germany

**Keywords:** bovine meat and milk factors, diet, colorectal cancer, chronic inflammation, macrophages, indirect carcinogenesis, myeloid immune checkpoint

## Abstract

Red meat consumption is considered a risk factor for colorectal cancer (CRC) development and stimulated isolation of plasmid-like DNA molecules from bovine serum and milk, termed bovine meat and milk factors (BMMFs). BMMFs encode a conserved replication protein (Rep). Increased populations of Rep-expressing macrophages have been identified in the peritumor of CRC patients and pre-cancerous tissues when compared to the tissues of healthy individuals. This supports the concept that BMMFs increase cancer risk by indirect carcinogenesis, upon induction of chronic inflammation. However, the spread of Rep^+^ immune cells in tissues at greater distances from primary tumors has not yet been assessed. Here, we immunohistologically analyzed the presence of Rep^+^ immune cells in sets of tumor, peritumor and, additionally, distant tissues of CRC patients (n = 13). We identified consistently high numbers of BMMF-positive macrophages in mucosal tissues at distances of as much as 25 cm away from the primary tumors, at levels comparable to peritumors and associated with M2-like macrophage polarization. The broad distribution of BMMFs suggests that BMMF^+^ macrophages might already exist at stages of pre-cancerous dysplasia or before. Quantification of BMMF tissue expression during colonoscopy might help to preventively stratify individuals at risk of developing polyps/CRC and recommend them for enhanced surveillance and/or changes in dietary lifestyle.

## 1. Introduction

Colorectal cancer (CRC) is the third most common cancer (constituting approximately 10% of all cancers) and the second leading cause of cancer deaths worldwide [1,2]. More than 1.9 million new cases and 930,000 deaths from CRC were recorded in 2020 worldwide, with the highest incidences in Europe, Australia and New Zealand and the highest mortality in Eastern Europe. It is estimated that numbers of new CRC cases and cancer deaths worldwide will increase by 63% (3.2 million cases per year) and 73% (1.6 million deaths per year), respectively, until 2040 [2]. The incidence of CRC remains high, even in high-income countries, benefitting from growing acceptance and capacity for CRC screening by colonoscopy. CRC risk intensifies with age, with most cases occurring in individuals older than 50 years. However, the number of individuals diagnosed with CRC under the age of 50 years, so-called early onset colorectal cancer, is rising worldwide [3]. Lifestyle factors contribute to the development of CRC: most notably, these include increased intake of processed meat, sedentary lifestyle, obesity, smoking, alcohol intake, etc. [2]. Epidemiological studies indicate a correlation between high consumption of bovine meat and milk products and regional incidence of certain cancers, most notably breast and colon cancers [4].

Bovine meat and milk factors (BMMFs) represent a steadily growing group of episomal, plasmid-like DNA molecules that are described as cancer risk factors, which were originally isolated from bovine sera and milk and, more recently, also from CRC and renal cancer tissues and detected in silico in NGS data [4,5,6,7]. In our laboratory, more than 180 different types of BMMFs have been isolated from bovine milk and serum and human tissues (overview in [5]), and, elsewhere, also from other species and other origins [8,9,10,11]. Immunohistochemistry based on antibodies against a conserved BMMF protein (Rep) [4] has revealed increased BMMF protein expression in the interstitial lamina propria of peritumors from CRC patients (tissue micro arrays, n = 246) as well as from cancer-preceding high- and low-grade dysplastic tissues, when compared to tissues of non-cancer individuals [4,12]. In all cases, Rep expression was associated with elevated levels of macrophages and detection of a surrogate marker for radical formation and oxidative DNA damage (8-Oxo-2′-deoxyguanosine, 8OHdG) in tissues close to the tumor (peritumor). This suggests a function of BMMF as a possible cancer marker and indicates a causal role of BMMFs in inflammation-driven indirect carcinogenesis of CRC, as outlined previously [4,12]. Increased peritumor expression levels have also been associated with a trend for decreased CRC-specific survival.

Although these data indicate both a possible predictive and prognostic relevance of peritumor BMMF-positive macrophage populations, we do not understand their spread in the remainder of the colon mucosa that is more distant from the primary tumor. Macrophages present in the tumor microenvironment are often more generally referred to as tumor-associated macrophages (TAMs), of which macrophages of the M2 type, in particular, have been associated with poor prognosis in many types of cancer [13,14,15]. In CRC, their role is less clear. However, more recent data have supported a CRC-promoting role of CD163^+^ (M2-like) macrophages, in general, or even of more uniquely described macrophage populations present in the tumor margin or close by [16,17,18]. In this study, we identified high BMMF Rep expression levels in predominantly M2-like macrophages in tissues surrounding the primary tumors, at distances of up to 25 cm. This indicates that BMMF expression is widely spread over the colon mucosa and might allow early identification of individuals at risk for tumors and pre-cancerous polyps during colonoscopy, even in situations where patients have not been diagnosed with defined tumors or polyps.

## 2. Materials and Methods

### 2.1. Selection of Patients and Medical Factors

All patients were treated by surgery at the Department of Surgery of University Hospital Mannheim (UMM), Germany, with written informed consent given by all participants. The study was conducted in accordance with the principles outlined in the Declaration of Helsinki (1975, revised in 2013) and approved by the ethics committee of the University of Heidelberg (vote 2012-293N-MA, approved 20 June 2012 and vote S-317/2023, approved 18 July 2023). The study was based on a randomized selection of samples based on gender (5 females, 4 males, 4 not available) and age (26 to 77 years, mean 63 years). Medical data were extracted from the hospital’s clinical documentation system (UMM). All diagnoses were reviewed by trained pathologists. No subjects were excluded from our study. An overview of patient metadata is provided in Table 1.

### 2.2. Human Tissue Collection and Preparation

Fresh tissues were taken from CRC patients (N = 13) after surgery at University Hospital Mannheim, in accordance with the local regulations, by trained clinicians. Tissue sizes were 1–1.5 cm and they were rinsed 3 times with PBS. Resected specimens were fixed in 4% formaldehyde (ROTI ^®^Histo-fix, Carl Roth GmbH, Karlsruhe, Germany) at room temperature overnight to prepare formalin-fixed paraffin-embedded (FFPE) tissue sections of 3 µm thickness using a Rotary Microtome HM 355S (Microm, Walldorf, Germany). They were then mounted on Superfrost Plus Adhesion Microscope Slides (Epredia, Breda, The Netherlands) for immunobiological analysis (in cooperation with the Light Microscopy Facility, DKFZ, Heidelberg, Germany), as described previously [7]. The analysis was conducted on tissue sets/sections comprising tumor, peritumor (max. 2 cm away from the primary tumor) and tumor-distant (at 12 cm, 17 cm or 25 cm distance from the primary tumor) tissues for each of the 13 individual donors.

### 2.3. Histology

FFPE-fixed tissue sections were used for IHC DAB (Diaminobenzidine) and immunofluorescence (IF) staining, whereby graded ethanol series and EDTA were employed for antigen retrieval (98 °C, 30 min), followed by primary antibody incubation (RT, overnight) and subsequent secondary antibody incubation (RT, 1 h). Bright field DAB (Diaminobenzidine, Zytomed DAB Substrate Kit, High Contrast, Berlin Germany) or fluorescence (Alexafluor dyes: goat anti-mouse Alexa Fluor 594, Invitrogen, Waltham, MA, USA, #A11032, 1:100; goat anti-rabbit Alexa Fluor 488, Invitrogen, Waltham, MA, USA, #A11034, 1:100; and donkey anti-mouse Alexa Fluor 647, Invitrogen, Waltham, MA, USA, #A31571, 1:100) detection reagents were used, as described previously [4,12]. Mouse monoclonal anti-Rep antibodies (AB3 and 10, 1:250, targeting BMMF1 group H1MSB.1, deposited and publicly available at the Leibniz-Institute German Collection of Microorganisms and Cell Cultures, DSMZ, Braunschweig, Germany) [4], anti-CD68 antibody (1:750, rabbit, #76437, D4B9C, Cell Signaling, Danvers, MA, USA) and anti-CD163 antibody (1:200, Novusbio, Centennial, CO, USA, NB110-40686) were applied for detection of BMMFs and macrophages, as described previously [12]. IgG1 (AB2536774, Invitrogen, Waltham, MA, USA) isotype control antibody was used for negative control staining. Stained slides were scanned with a Hamamatsu NanoZoomer S60 slide scanner (version 2.0-HT, Hamamatsu Photonics, Hamamatsu, Japan).

### 2.4. Quantification Analysis of Immunofluorescence Staining

Cell-based quantification on digitalized scans of co-immunofluorescence (IF) staining of Rep^+^ and CD68^+^ and CD163^+^ macrophages was performed with a script based on DAPI nuclear and antibody staining based on Fiji (v1.52, open source) [19] of 4–6 randomly selected microscopic areas, as previously described (around 5000 cells quantified, in total, per patient) [12]. The data were represented as % of Rep^+^, CD68^+^ or Rep^+^CD68^+^ double and Rep^+^CD68^+^CD163^+^ triple positive cells with respect to total cells quantified in the interstitium (consistently excluding crypt cells).

### 2.5. Statistical Analysis

Paired (two-sided) *t*-tests were used for statistical analysis of different tissue regions from the same individuals. Correlation analysis of Rep and CD68 staining in different tissue regions was determined by Pearson rank correlation coefficient rho (GraphPad Prism, RRID:SCR_002798, V9.3.0, GraphPad Software Inc., Boston, MA, USA). In general, *p* > 0.05 indicates no significant difference, while *p* ≤ 0.05 shows a significant difference, indicated by * *p* ≤ 0.05, ** *p* ≤ 0.01, *** *p* ≤ 0.001, **** *p* ≤ 0.0001.

## 3. Results

### 3.1. BMMF Rep Expression Is Detectable in Mucosa Far Distant from Primary CRC Tumors

**Figure 1:**
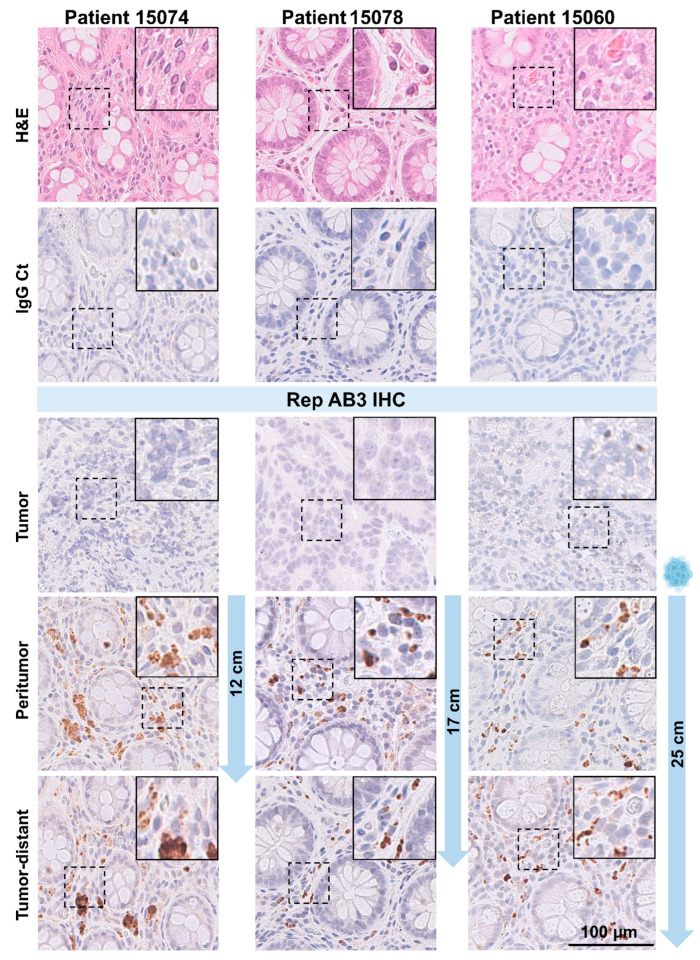
Immunohistochemical detection of BMMF Rep protein expression in different tissue parts of CRC patients. Hematoxylin and eosin (H&E) and IHC DAB staining of FFPE tissue sections with anti-IgG1 (isotype control), anti-Rep (AB3) antibodies of tumor, peritumor and tumor-distant tissues (taken at 12, 17 or 25 cm distance from primary tumors) from 3 different representative CRC patients (representation of IgG isotype control staining: tumor distant tissue, scale bar: 100 μm, magnification of enlargements: 2×).

**Figure 2:**
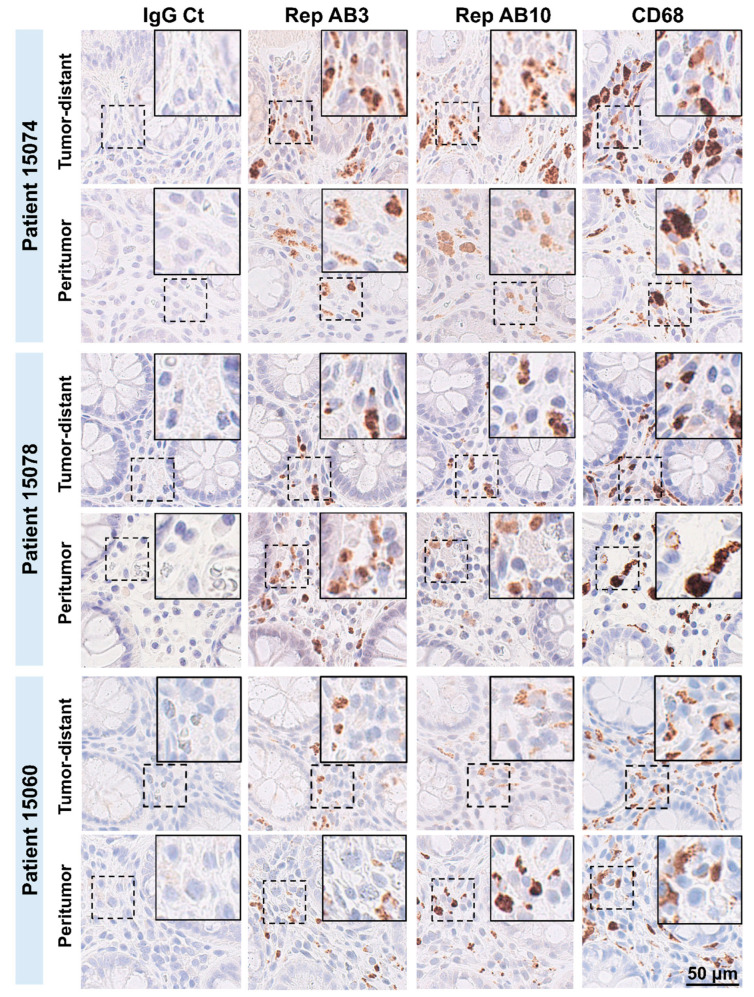
Immunohistochemical detection of BMMF Rep and CD68 in peritumor and tumor-distant tissues of CRC patients. IHC DAB staining with anti-Rep antibodies AB3 and AB10 or anti-CD68 antibodies applied on consecutive FFPE tissue sections of tumor-distant or peritumor tissue of 3 representative CRC patients (IgG Ct: isotype control, scale bar: 50 μm, magnification of enlargements: 2×).

Previous studies based on semi-automated immunohistochemical staining with anti-BMMF Rep antibodies showed increased BMMF Rep expression in the peritumor tissues of CRC patients compared to the tumor and colonic tissues of individuals without cancer. Here, we expanded our analysis and not only focused on tumor and peritumor (max. 2 cm away from the primary tumor), but also assessed Rep staining in tissues located at larger distances from the primary tumor (up to 25 cm away), allowing description of a metric distribution of Rep expression in CRC patients. Therefore, tumor, peritumor and distant tissue regions were used for preparation of FFPE tissue sections from a total of 13 individual colorectal cancer patients and subsequently subjected to immunohistochemistry (IHC) with anti-Rep antibody AB 3 [4]. Distant tissues were prepared at the following distance from the primary tumor: 12 cm (n = 4 patients), 17 cm (n = 5) and 25 cm (n = 4). As expected and reported previously, Rep expression was detected in all peritumor tissues in the interstitium, between the crypts of Lieberkühn, but not observable (or only to a very low degree) in the associated tumor tissues. Regarding Rep staining in the associated tissues taken at larger distances from the primary tumors, all tissues showed intense tissue staining, comparable to the staining observed for the peritumor tissues (Figure 1). Control staining with isotype control antibodies (IgG1 Ct) on consecutive sections did not reveal any distinct staining, which supports the specificity of the staining. There was no visible difference in staining intensity between paired tumor-distant and peritumor tissues, or among tumor-distant tissues prepared at different distances from the primary tumors (12 cm, 17 cm and 25 cm, as shown by columns 1 to 3). IHC staining based on a second anti-Rep antibody (AB 10) [4] revealed similar staining intensity when compared to AB 3, which is consistent with previous findings. IHC staining with both anti-Rep AB3 and AB 10 consistently stained comparable tissue foci in the lamina propria, between the crypts of Lieberkühn, and close to cells with smaller sized nuclei in both tumor-distant and peritumor tissues (Figure 2).

### 3.2. BMMF Rep Expression Is Associated with CD68^+^ Macrophages in Both Peritumor and Distant Tissues

To test the association of Rep staining and macrophages (MPs), IHC DAB staining was performed on consecutive cuts, whereby an anti-CD68 antibody was used as marker for MPs. In general, very similar Rep and CD68 staining patterns were observed in tumor-distant and peritumor tissues (Figure 2). As observed for Rep staining, IHC staining for anti-CD68 revealed no visible difference in macrophage staining intensity between paired tumor-distant and peritumor tissues, or among tumor-distant tissues taken at different distances from the primary tumors.

**Figure 3:**
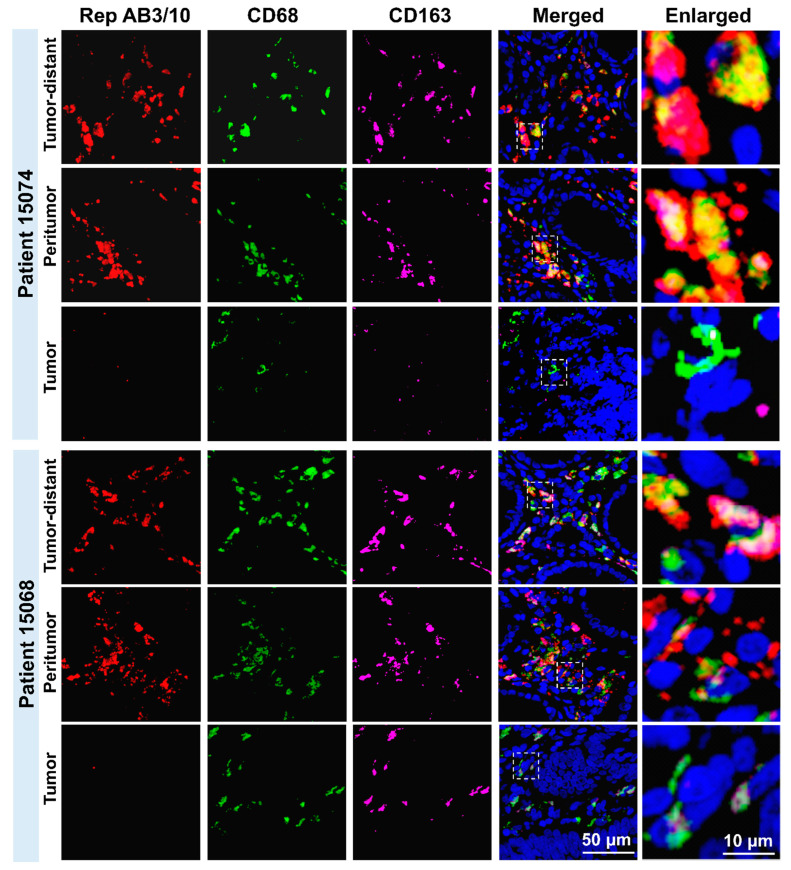
Co-immunofluorescence (IF) staining of Rep^+^, CD68^+^ and CD163^+^ cells in tumor, peritumor and tumor-distant tissue regions of CRC patients. Representative images of IF staining for DAPI (blue), anti-Rep (AB3 and 10, red), anti-CD68 (green) and anti-CD163 (purple) of tumor-distant, peritumor, and tumor tissue from 2 representative CRC patients (scale bar: 50 μm, magnification of enlargements for dashed selection frames: 5×).

We next performed co-immunofluorescence (IF) staining and cell-based quantification for Rep, CD68 and CD163 (a marker of M2-like MPs) to analyze the association of Rep expression with the number of macrophages in more detail. IF staining showed a strong, predominantly cytoplasmic and congruent staining of Rep (red), CD68 (green) and CD163 (purple) in the interstitial lamina propria (between the crypts of Lieberkühn), both for peritumor and distant tissues (Figure 3). In agreement with the IHC DAB staining and previous data, there was negligible Rep detection in the tumor.

**Figure 4:**
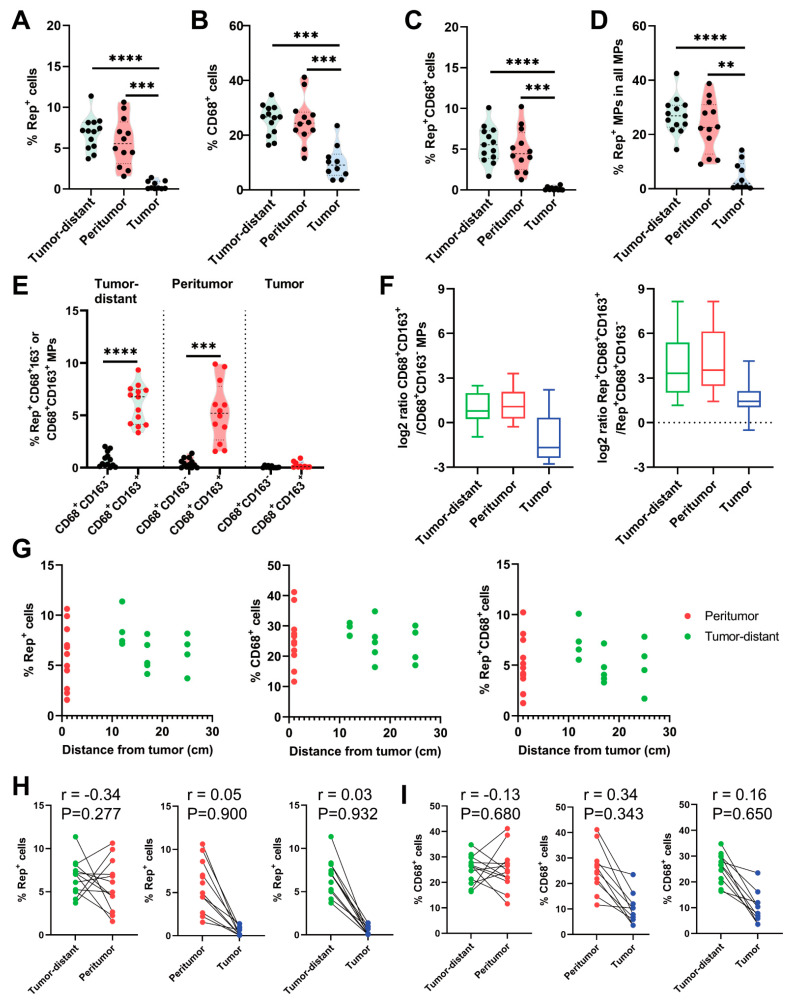
Quantification of Rep/CD68/CD163-positive cells by co-immunofluorescence staining in tumor-distant, peritumor and tumor tissues. Quantification of the number of Rep^+^ cells (% of all interstitial cells) (**A**), CD68^+^ macrophages (% of all interstitial cells) (**B**), Rep^+^CD68^+^ macrophages (MPs) (% of all interstitial cells) (**C**) and Rep^+^CD68^+^ MPs (% with respect to interstitial MPs) (**D**). (**E**) Quantification of Rep-positive M2-like (CD68^+^CD163^+^) or non-M2-like (CD68^+^CD163^-^) macrophages (% with respect to all interstitial cells). (**F**) Log2 ratio of M2-like vs. non-M2-like macrophages (left) and Rep-positive M2-like vs. non-M2-like macrophages (right) (error bars indicating maximum/minimum). (**G**) Comparison of Rep^+^ cells, CD68^+^ MPs and Rep^+^CD68^+^ MPs (% with respect to all interstitial cells) in peritumor and tumor-distant tissues of 13 different CRC donors. Tumor-distant tissues were resected in regions at 12 cm (n = 4), 17 cm (n = 5) or 25 cm (n = 4) distance from the primary tumor. Correlation of Rep^+^ cells (**H**) and CD68^+^ macrophages (**I**) within pairwise comparisons of tumor, peritumor or tumor-distant tissues (Pearson’s correlation). Paired (two-sided) *t*-test used for statistical analysis of different tissue regions from individuals—tumor-distant (n = 13), peritumor (n = 12) and tumor (n = 10). ** *p* ≤ 0.01, *** *p* ≤ 0.001, **** *p* ≤ 0.0001.

Quantification of IF staining revealed an increased number of Rep^+^ cells (% with respect to total cells) in tumor-distant (mean 6.8%) and peritumor (5.8%) tissues of CRC patients compared to tumor tissues (0.5%, *p* < 0.0001 and *p* < 0.001, respectively; Figure 4A). Additionally, a higher number of CD68^+^ macrophages was detected in tumor-distant (25.6%) and peritumor tissue (25.2%) compared to tumor tissues (10.0%, *p* < 0.001 and *p* < 0.001; Figure 4B). Between tumor-distant and peritumor tissues, the number of Rep^+^ cells did not vary significantly (*p* = 0.457; Figure 4A). Also, numbers of CD68^+^ macrophages were at a similar level (*p* = 0.985; Figure 4B).

Quantification of Rep^+^CD68^+^ macrophages resembled quantification of Rep^+^ cells alone, and numbers were increased in tumor-distant (mean 5.6%) and peritumor (4.9%) tissues compared to tumor tissues (0.2%, *p* < 0.0001 and *p* < 0.001; Figure 4C). There was no significant difference in the numbers of Rep^+^CD68^+^ double positive cells between tumor-distant and peritumor tissues (*p* = 0.642; Figure 4C). As we focused on populations of Rep-positive MPs within the population of MPs, specifically, we noticed that Rep-positive MPs represent up to 40% of all detected MPs in peritumor and tumor-distant tissues, with means of 23.1% in peritumor and 27.0% in tumor-distant tissues (Figure 4D). Fractions of Rep^+^CD68^+^ MPs among all MPs were increased in tumor-distant (mean 27.0%) and peritumor (23.1%) tissues of CRC patients compared to tumor tissues (4.7%, *p* < 0.0001 and *p* < 0.01). No significant difference was observed between tumor-distant and peritumor tissues (*p* = 0.228; Figure 4D).

### 3.3. BMMF Rep Expression Is Associated with an M2-like Macrophage Polarization

Next, we characterized polarization of myeloid immune cells either toward M2- or non M2-like MP polarization, together their association with Rep detection by triple IF staining with anti-Rep, anti-CD68 and CD163 antibodies, as described previously [12]. Detection of Rep^+^ cells was more frequently associated with M2-like (CD68^+^CD163^+^) MPs, as demonstrated by an increased number of Rep^+^CD68^+^CD163^+^ triple positive cells in comparison to Rep^+^ non-M2-like MPs (Rep^+^CD68^+^CD163^−^ cells) in both tumor-distant (mean 6.1% vs. 0.7%, *p* < 0.0001) and peritumor tissues (5.3% vs. 0.5%, *p* < 0.001; Figure 4E). This is also reflected by increased log2 M2/non-M2 ratios of Rep-positive MPs (Figure 4F, right) compared to log2 M2/non-M2 ratios for MPs alone (left).

### 3.4. Broad Distribution of BMMF Rep and Macrophages in Tumor-Distant Tissues of CRC

Based on the IHC DAB and IF staining, we noted comparable levels of Rep and CD68 tissue staining in tumor-distant and peritumoral tissues. We next assessed metric distribution of tissue staining in more detail by associating staining intensity with the sampling distance to the tumor. We observed equally high numbers of Rep^+^, CD68^+^ and Rep^+^CD68^+^ cells at all three distances (12, 17 and 25 cm) (Figure 4G). Correlation analysis comparing numbers of Rep^+^ cells per patient in peritumor and tumor-distant tissues revealed no significant associations (Figure 4H, left). Comparison of peritumor with tumor, as well as of tumor-distant with tumor tissues, also did not reveal any significant associations regarding the quantity of Rep^+^ cells per patient (Figure 4H, middle and right). The same trends (but at generally higher detection levels) were observed for pairwise comparison of the numbers of MPs in individual CRC donors (Figure 4G).

## 4. Discussion

### 4.1. BMMF Expression Is Widely Spread in the Mucosal Tissues of CRC Patients

**Figure 5:**
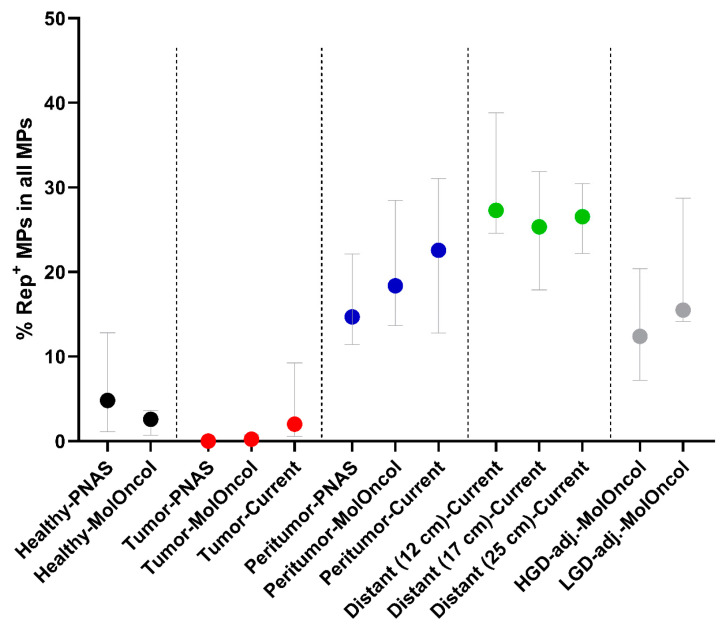
Summary of the fraction of BMMF Rep-positive macrophages among all macrophages identified in previous publications, labeled ‘PNAS’ [4], ‘MolOncol’ [12] and in the present study (‘Current’) (data presented as medians with interquartile range). Tissues were from healthy non-cancerous individuals (black, n = 8 and 10 donors, respectively), CRC tumors (red, n = 7, 26, and 10, respectively), CRC peritumors (blue, n = 7, 26, and 12, respectively), tissues distant to CRC primary tumors (green, n = 4, 5 and 4, respectively) or tissues adjacent to high- or low-grade dysplasia (grey, HGD, LGD, n = 14 and 9, respectively).

BMMFs are a fast-expanding class of small plasmid-like DNA elements that were initially and are mainly identified in bovine meat and milk products [5]. The amount of macrophages showing expression of a conserved BMMF protein (Rep) was significantly increased in the peritumor mucosa of CRC patients, when compared to mucosal tissues of age-matched cancer-free individuals [4,12] (Figure 5). As Rep expression was also elevated in tissues surrounding high- and low-grade dysplasia, it was concluded that BMMFs might serve as marker for (early) cancer detection and, additionally, might also causally contribute to the development of CRC via indirect carcinogenesis, by promoting chronic tissue inflammation over long periods (Figure 5). These crucial findings are supported by identification of increased levels of tumor-promoting CD68^+^CD163^+^ M2-like MPs, together with Rep expression, as well as increased detection of markers for oxidative DNA damage (8OHdG), most probably evoked by inflammation-driven radical formation. These radicals diffuse and reach replication-competent stem cells and early daughter cells in the colonic crypts and promote random DNA mutation and, finally, mutations in cancer driver genes, thus turning these cells into cancer progenitor cells [4,12]. Lack of recruitment of T- or B-cells to these tissue areas supports the overall assumption that this state of tumor-promoting tissue inflammation is steered by myeloid immune cells that are causally involved in carcinogenesis. The limited survival of CRC patients with high levels of BMMF Rep^+^ MPs also infers a prognostic relevance of this peritumor deregulation, suggesting the presence of a myeloid immune checkpoint with both causal (predictive) and prognostic relevance for CRC development and outcomes.

Unfortunately, the spread and dimensions of such BMMF Rep^+^ (myeloid) immune cell populations, which might promote such causal and prognostic functions, have not been yet assessed; in addition, analyses were limited to the tissue in the direct vicinity of primary CRC tumors (at up to 2 cm distance) or the tumor and dysplastic tissues themselves. Thus, it is not clear whether the spread of BMMF-positive myeloid immune cells only affects the direct microenvironment of tumor and pre-cancerous dysplasia, or larger areas of the colon mucosa. Here, we observed that expression of BMMF Rep is also detectable in CRC patients’ colon mucosa at greater distances, of up to 25 cm away from the primary tumors. This observation significantly expands previous data which describes that CD68 and CD163 positive MPs were detected at maximum quantities closer to the tumor nests (i.e., in and closely adjacent to the invasive margin) [16,18,20]; however, these studies did not focus on systematic quantification in tissues at larger distances from the tumor nests. Rep expression was still almost exclusively associated with macrophages occurring at the same location (cytoplasmic speckles) and intensity, as observed in peritumor tissues in both this and previous studies [4,12]. Intensity of Rep expression and numbers of Rep-positive cells were high in mucosal tissues of CRC patients, independent of the sampling position at 12, 17 or 25 cm distance from the primary tumor. MPs, in general, were detected at comparably high levels throughout all mucosal tissue locations.

### 4.2. BMMF^+^ MPs Could Represent a Widespread Immune Risk Signature in CRC and Pre-Cancer Stages

Detection levels of MPs, Rep^+^ cells or Rep^+^CD68^+^ MPs did not vary with the different distances, and were equally high. Importantly, the location of the tissue in the either ascending, traversal, descending or sigmoidal colon also did not seem to affect the homogeneous (high) distribution of Rep staining, either in this limited exploratory study or in previous studies [12]. This indicates that formation and conservation of this myeloid BMMF signature might rely on very widespread effects involving BMMF uptake, possible migration and regulation, which were not assessed here but must be studied in future. This also means that events like formation of early and late dysplasia, as well as final formation of adenocarcinomas/tumors, are embedded in a rather homogeneous tissue background of BMMF-positive myeloid cells. Because detection of Rep was significantly associated with a M2-like polarization of MPs (CD68^+^CD163^+^) over all distances tested, this widespread tumor-promoting immune landscape might represent a very broad—not locally limited—risk signature and biomarker for CRC, which is available for testing throughout the entire mucosa. The pro-tumorigenic role of CD163^+^ M2-like MPs has previously been described by several studies of CRC [16,21,22] including CRC mouse models [23], and other types of cancer [15,24,25]. In our previous studies, tissues adjacent to early- and late-stage dysplasia, which in >90% of cases precede CRC tumors, were already found to show significantly increased levels of Rep^+^CD68^+^CD163^+^ MPs when compared to mucosal tissues of cancer-free individuals (Figure 5). Together with the broad and invariant spread of the BMMF myeloid landscape observed in this study, this supports the hypothesis that such a widespread cancer-promoting BMMF myeloid immune landscape might already exist in individuals with early- and late-stage dysplasia, and, most likely, even in individuals without dysplasia but at high risk of developing dysplasia. Reasons for this might be a generally higher uptake of BMMFs and/or decreased capabilities for clearing incoming BMMF components or BMMF-positive myeloid cells in these compromised individuals. Additionally, immune electron microscopy with anti-Rep antibodies has revealed frequent occurrence of pleomorphic vesicular bodies in peritumor tissues of CRC, lung and pancreatic cancer patients [7]. This further suggests widespread patterns of BMMF^+^ immune cell populations and indicates a dynamic and flexible route of transmission.

### 4.3. Potential for Prospective Cancer Screening During Colonoscopy

Presence of a widespread, BMMF-linked and possibly pathogenic high-risk myeloid immune landscape could herald the following prospects: (i) diagnostic detection of CRC by testing for BMMF expression in peritumor tissues, or any other biopsy, (ii) early detection of high-risk dysplasia by testing for BMMF expression in tissues adjacent to dysplasia, or any other biopsy, during colonoscopy, (iii) early detection of a high-risk BMMF immune landscape preceding polyp formation in any biopsy material, during colonoscopy. Currently, colonoscopy represents the first line of CRC prevention and is suggested at the age of 50 for males and 55 for females [26]. Efficacy of colonoscopy is mainly based on (early) identification and characterization of polyps (early- and late-stage dysplasia), and tumor prevention largely relies on resection of the cancer-preceding polyps. However, successful prevention is limited by the fast growth of undetected polyps, which might develop unnoticed between screening intervals and not include stratification of any high-risk immune signatures or other biomarkers. Inclusion of BMMF biomarkers into such procedures would maximize the full potential of current colonoscopy, providing new possibilities for early detection of CRC and, thus, for primary and secondary cancer prevention. Individuals stratified as ‘high CRC risk’ upon increased detection of BMMF^+^ MPs could be recommended for enhanced surveillance and/or changes of dietary lifestyle, to a low inflammation diet, avoiding e.g., BMMF-containing bovine meat and milk.

### 4.4. Limitations of the Study

This exploratory study would benefit from cohort expansion and higher resolution for different locations within the large intestine (including samples taken at different distances from the tumor of single patients), to overcome experimental variations due to the high biological variability among and within individuals and inclusion of biopsies taken at distant positions to sites of dysplasia. Those samples could be compared with biopsies from non-cancerous/non-dysplasia-experiencing donors to test for the feasibility of early detection by analyzing BMMF tissue expression, including discriminators such as, e.g., clinical background, diet, genetic/familiar predisposition, and immune-modulatory treatments. This current study cannot support mechanistical conclusions or proof-of-concept for any causal or pathogenic involvement of BMMF in carcinogenesis, which must be assessed with prospective screening studies (including, e.g., serology) and/or models to test BMMF-specific tumor induction, utilizing, e.g., BMMF exposure in mouse models (e.g., with a mild baseline tumor induction, such as heterozygous Apc). Future studies would also benefit from inclusion of additional BMMF markers, as well as the use of specimens of other types of putative BMMF-positive cancers, such as tissues from, e.g., liver, pancreas or lung cancer patients.

## 5. Conclusions

Despite the awareness of several risk factors for colorectal cancer (CRC), including hereditary factors or diet-linked factors like red meat consumption, not much is known about the molecular presence of any agents involved or their mode of action. Bovine meat and milk factors (BMMFs) are plasmid-like DNA molecules that are frequently isolated from bovine meat and milk. BMMFs replicate and encode for BMMF proteins (Rep) in human cells. Increased numbers of BMMF Rep-positive macrophages were observed in tumor-adjacent tissues of CRC patients compared to healthy individuals. Therefore, BMMFs were suggested as cancer risk factors, contributing to the development of CRC via chronic inflammation-linked indirect carcinogenesis and DNA damage. Here, by systematic immunohistochemical quantification of BMMF tissue expression, we identified a widespread distribution of BMMF-positive myeloid immune cells, which were not only limited to the direct vicinity around the primary tumor but also present in the mucosa far away from the tumors (>25 cm). The BMMF-positive myeloid immune cells consistently displayed polarizations closer to those of M2-types of macrophages, for which a tumor-promoting role in CRC has become more and more evident in recent literature. These findings suggest that large numbers of cancer-promoting BMMF-positive macrophages might be spread over extended parts of the colonic mucosa and that—in combination with previous findings—they might already exist at times of pre-cancerous dysplasia or even before that. This offers the option to test for such potentially pathogenic myeloid immune cells during colonoscopy procedures. Thus, individuals at risk of displaying any tumor-promoting BMMF-linked immunologic phenotype could be identified and recommendations made for enhanced surveillance and/or changes in lifestyle/diet.

## Figures and Tables

**Table 1:** Overview of patient clinical information.

		Paired Tissues	
		N	%
Gender	Male	4	38.5
	Female	5	30.8
	not available	4	30.8
	Total	13	100.0
Age	<50	2	15.4
	50–59	12	92.3
	60–69	2	15.4
	70–79	6	46.2
	not available	4	30.8
	Average	63	100.0
Localization	Ascendens	1	7.7
	Sigmoid	5	38.5
	Cecum	2	15.4
	not available	5	38.5

## Data Availability

The data that support the findings of this study are available from the corresponding author upon request.

## Abbreviations

The following abbreviations are used in this manuscript:

AB	Antibody
BMMFs	Bovine meat and milk factors
CRC	Colorectal cancer
IF	Immunofluorescence
IHC	Immunohistochemistry
MPs	Macrophages
NGS	Next generation sequencing
Rep	Replication protein
